# Smoking cessation in inpatient psychiatry treatment facilities: A review

**DOI:** 10.1016/j.abrep.2020.100255

**Published:** 2020-01-30

**Authors:** Robert Kagabo, Adam J. Gordon, Kola Okuyemi

**Affiliations:** a Department of Family and Preventive Medicine, University of Utah School of Medicine, Salt Lake City, UT, United States; b Program for Addiction Research, Clinical Care, Knowledge and Advocacy (PARCKA), Division of Epidemiology, Department of Internal Medicine, University of Utah School of Medicine, Salt Lake City, UT, United States; c Informatics, Decision-Enhancement, and Analytic Sciences (IDEAS) Center, VA Salt Lake City Health Care System, Salt Lake City, UT, United States

**Keywords:** Smoking Cessation, Inpatient Settings

## Abstract

**Background:**

Tobacco-related diseases are a leading cause of death among individuals with severe mental illness (SMI), yet interventions to address tobacco cessation are rare in inpatient settings where persons with SMI are hospitalized. While cigarette smoking rates have declined in the general population, they remain high in persons with SMI. Inpatient settings would be a prime location to intervene on tobacco consumption among persons with SMI. The objective of this review was to examine evidence of smoking cessation interventions in psychiatric inpatient facilities.

**Method:**

Using narrative overview guidelines, we searched PubMed, PsycINFO, and CINAHL for smoking cessation RCT studies published between 1950 and 2018. Studies included had to have at least started in inpatient psychiatry settings. Examples of search terms included: smoking cessation in inpatient psychiatry, smoking cessation in inpatient mental health treatment facilities, and smoking cessation and mental health.

**Results:**

Following the inclusion criteria, eight RCT studies were reviewed. One study was among adolescent psychiatric inpatient smokers ages 13–17, and 7 were among adult psychiatric inpatients with mean age 41 years. Treatment periods lasting 8 to 12 weeks started in inpatient settings and continued post discharge. A combination of behavioral and pharmacological interventions were used. Pharmacological interventions were nicotine replacement therapies, and at least one study used varenicline. At baseline, participants smoked an average of 18.1 cigarettes per day.

**Conclusion:**

Smoking cessation in inpatient psychiatry settings is rare or delayed. There is a need for more tailored treatments among this population to help them quit smoking.

## Highlights

• Smoking rates are high among people with psychiatric illness.
• Smoking cessation interventions are rarely available in inpatient psychiatry settings.
• Smokers with psychiatric illness are just as interested in quitting smoking.

## 1 Background

Tobacco-related diseases such as cancers, heart disease, and lung diseases are the leading cause of death among individuals with mental illness (Colton and Manderscheid, 2006, Prochaska, 2010). In addition, individuals with mental illness face tobacco-related isolation, stigma, or financial hardships (Prochaska, Hall, & Hall, 2009). Tobacco use is also reported to increase the metabolism of some psychiatric medication (Prochaska et al., 2009). While it is estimated that individuals with mental illness make up only 22% of the United States’ population, estimates are that each year 200,000 of the 443,000 tobacco-related deaths are among individuals with mental illness (Schroeder and Morris, 2010, Williams and Ziedonis, 2004). Smoking cessation programs are however rare in inpatient psychiatry and substance use treatment facilities including in facilities where smoking is not allowed (Prochaska et al., 2004, Prochaska et al., 2014). The objective of this review was a narrative overview or examination of evidence of smoking cessation interventions in psychiatric inpatient treatment facilities.

The historical culture of permitting smoking in psychiatry units has led to more than half of psychiatry facilities in the US permitting smoking in their units (Prochaska, Hall, Delucchi, & Hall, 2014). Individuals with mental illness are more likely to smoke compared to the general population and their smoking rates remain consistently high (Metse et al., 2017, Szatkowski and McNeill, 2015). Up to 85% of individuals with mental illness are current cigarette smokers (Kalman et al., 2005, Leonard et al., 2001, McClave et al., 2010), a rate that is 4 times higher than the general population (CfDCaP, 2008, McClave et al., 2010). Other studies reported that 60% of adolescents in inpatient psychiatric care were current smokers with 40% smoking at least a pack of cigarettes each day (Prochaska et al., 2013, Ramsey et al., 2002). While there have been declining smoking trends in the general population, such has not been observed among people with psychiatric illness (Cook et al., 2014, Szatkowski and McNeill, 2015). Some research has shown that when there is a failure to address tobacco dependence, majority of psychiatric patients who quit smoking as inpatients relapse following their hospital discharge, and unaddressed nicotine dependence compromises psychiatric care (Prochaska et al., 2004, Prochaska et al., 2009). Given such high rates of smoking and the difficulty to quit, there is a need to understand how smoking cessation interventions work, and are implemented among this population of individuals with psychiatric illness in inpatient settings.

## 2 Smoking in inpatient psychiatry settings

Smoking has been a widely accepted culture in psychiatry units including in some cases clinicians smoking with patients (Brown-Johnson et al., 2014, Dickens et al., 2004). The Joint Commission which is the entity responsible for accreditation and certification of US healthcare Organizations’ mandate in 1992 for hospitals to ban smoking in the United States was met with so much advocacy for patients’ need and right to smoke (Brown-Johnson et al., 2014). Such advocacy and outcries later led the Joint Commission to exempt psychiatry units from the smoking ban (Brown-Johnson et al., 2014, Lawn and Pols, 2005, Taylor et al., 1993). In most cases, cigarette smoking has been used to reward patients for good behavior in these psychiatry units or as a privilege to reinforce patients’ medication use compliance (Olivier, Lubman, & Fraser, 2007). Clinicians who smoke with patients within psychiatric inpatient facilities reported that smoking together is a good way to build clinician and patient rapport (Dickens et al., 2004, Prochaska et al., 2014). In addition, tobacco use treatment is ignored or delayed by many clinicians who do not view the dependence as a disorder in the area of mental illness treatment (Williams & Ziedonis, 2004). Other reasons for delayed treatment include lack of education or knowledge of association between smoking and psychiatric disorders, and nicotine dependence medical-related complications (Hughes and Frances, 1995, Rosen-Chase and Dyson, 1999).

## 3 Methods

This paper is a narrative overview of smoking cessation randomized control trial studies that started at least in psychiatry inpatient settings. We searched PubMed, PsychINFO, and CINAHL for articles of smoking cessation studies in inpatient psychiatry settings published between 1950 and December 2018. Search terms and phrases used included; smoking cessation in inpatient psychiatry, smoking cessation in inpatient mental health treatment facilities, smoking cessation and mental health, smoking cessation and substance use treatment, and tobacco treatment in inpatient psychiatry treatment facilities. The search terms also included smoking cessation clinical trials or randomized trials in inpatient psychiatry.

### 3.1 Inclusion and exclusion

The final journal articles included in this paper were those of randomized controlled trials that at least started in inpatient psychiatry settings and were published between 1950 and December 2018. Any studies that were not randomized controlled trials or did not start in inpatient settings were excluded. To avoid duplication, only one manuscript was reviewed from each randomized controlled study found during the searches. Studies of smoking cessation among outpatient settings were also excluded.

## 4 Results

Following the exclusion and inclusion criteria, a total of eight (8) peer reviewed journal articles were reviewed and included in this paper. Of the 120 articles found, 112 were excluded, and all included were randomized controlled trials. One study was among adolescent psychiatric inpatient smokers ages 13–17, and 7 studies were among adult psychiatric inpatients with mean age 41 years. A summary of the included studies is shown in Table 1. As indicated in the inclusion criteria, all the 8 studies included started at least when participants were in inpatient settings and lasted between 8 and 12 weeks. Treatment continued after discharge in some cases with follow up periods between 1 and 18 months. Of the controlled trials included in the review, 7 out of 8 used a combination of behavioral and pharmacological interventions, and one study had only pharmacological interventions. In 7 of the studies the pharmacological interventions were nicotine replacement therapies (NRT), and one study used varenicline as the pharmacological intervention. At baseline, participants smoked an average of 18.1 cigarettes per day, and up to 85% of individuals with psychiatric illness were current cigarette smokers. The exclusion criteria used by authors in the studies we reviewed were based on issues such as serious medical contraindications to NRT, or severe aggression during the hospital stay (Schuck et al., 2016). Additional exclusion criteria used by authors in the reviewed studies included participants being young than 18 years and not fluent in the English language (Prochaska et al., 2014).

**Table 1:** Summary of smoking cessation clinical trial studies in inpatient psychiatry and substance use treatment facilities.

Author (year published)	Outcome Measures	Setting	Population	Mean Age	Mean cigarettes per day	Treatment Type	Follow-up (months)	Sample size
Metse et al. (2017)	1-Month prolonged abstinence 7-day point prevalence abstinence	Started in inpatient psychiatric facilities and continued post-discharge	Inpatients at 4 psychiatric facilities in Australia	38.7		Usual care compared to brief MI plus 4-month pharmacological & psychosocial intervention post discharge	4, 6–12	754
Schuck et al. (2016)	Number of 24 h quit attempts	Started in Inpatient psychiatry	Adult inpatients from seven from seven psychiatric units at 3 San Francisco Bay Area hospitals.	39	17	Usual care compared to brief (3 months) treatment group, and extended (6 months) treatment group. Treatment groups received transtheoretical-model tailored computer intervention. NRT was given to participants	18	956
Smith et al. (2016)	Reduce smoking and improvement of cognition	A meaningful portion of the sample were inpatients	Patients with schizophrenia	45.1	17.6	Varenicline compared to placebo	8-weeks treatment	87
Hickman III et al. (2015)	7-day point prevalence abstinence	From three psychiatric units at San Francisco General Hospital. 10-week post hospital NRT	Adult smokers uninsured, indigent, unhoused	39.5	19	NRT + Advice compared to Transtheoretical – model tailored computer-assisted intervention		100
Stockings et al. (2014)	7-day point prevalence abstinence	Initiated in inpatient care and continued for 4-months post-discharge	Patient smokers at an inpatient psychiatric facility in Australia	37.6	23.0	Usual Treatment compared to psychosocial and pharmacological (NRT) support		205
Prochaska et al. (2014)	Verified 7-day point prevalence abstinence	Initiated in Inpatient Psychiatry and continued in outpatient treatment 10-week post NRT?	Patients in locked acute psychiatry unit at the Langley Porter Psychiatric Institute at the University of California		19.0	Usual care compared to (Motivational interviewing + NRT)	18-months NRT was provided for 10 weeks	224
Chen et al. (2013)	Smoking reduction, and 7-day point prevalence abstinence	Inpatients with schizophrenia and schizoaffective disorders	Two public inpatient psychiatry facilities in Taiwan	45.2	13.1	High dose NRT (31.2 mg) compared to Low dose NRT (20.8 mg)	8-weeks treatment	184
Brown et al. (2003)	Point prevalence abstinence, quit attempts, change in smoking rate, and longest quit attempt	Psychiatry inpatient	Adolescents at a private university hospital in Providence Rhode Island	13–17		Motivational Interviewing (MI) versus brief advice (BA). MI was two 45 min sessions and BA was 5–10 min advice with quit information. NRT was provided at discharge	1, 3, 6, 9, and 12	191

## 5 Discussion

### 5.1 Smoking cessation in psychiatric inpatient facilities

The goal of this review was an examination of existing evidence of smoking cessation interventions in psychiatric inpatient treatment facilities. In this review we found that smokers with psychiatric illness in psychiatric treatment facilities rarely receive smoking cessation treatment and effective services are needed to assist them quit (McFall et al., 2010). Hospitals offering inpatient services have been identified as opportunity settings to begin tobacco dependence treatment and as settings where smokers are motivated to quit or accept quitting messages (Bernstein et al., 2008, Dohnke et al., 2012). However, few studies reported initiation of smoking cessation treatments while participants were inpatients in psychiatric facilities and continued treatment for at least 4–6 months post-psychiatric hospital discharge (Hickman et al., 2015, Metse et al., 2017, Prochaska et al., 2014, Stockings et al., 2014). In some of these studies, psychiatric patient participants were recruited regardless of their intention to quit smoking (Barnett et al., 2015, Hickman et al., 2015). The limited research regarding smoking cessation among people with mental illness shows this group is just as interested in quitting and in a few cases have quit attempt rates similar to smokers without mental illness (McClave et al., 2010, Metse et al., 2017, Siru et al., 2009).

### 5.2 Tobacco use treatment

Like in the general population, smokers with psychiatric illness use both pharmacological and psychosocial strategies in their attempts to quit smoking (Metse et al., 2017, Schuck et al., 2016, Stockings et al., 2014). Results from clinical trials have shown smoking quit rates of these strategies to be between 4% and 22% among people with psychiatric illness (Banham and Gilbody, 2010, Barnett et al., 2015). In patients with schizophrenia disorders for example, bupropion and varenicline have been found to be effective pharmacotherapies for smoking cessation (Barnett et al., 2015, Evins et al., 2014, Tsoi et al., 2010, Williams et al., 2012). Treatment modalities that use a combination of psychosocial and pharmacotherapies are effective in increasing abstinence rates. In outpatient settings for example, depressed smokers who received cognitive-behavioral and nicotine replacement therapies doubled cessation rates (Hickman et al., 2015). On the other hand, smokers with schizophrenia who were treated with a combination of buproprion and nicotine replacement therapy (NRT) had more abstinence rates compared to schizophrenia smokers treated only with NRT (Evins et al., 2007, Hickman et al., 2015).

### 5.3 Psychosocial or behavioral intervention

Psychosocial or behavioral interventions included motivational interviewing (MI) and brief advice (BA). Brown et al. defined the motivational interviewing they used as two 45 min long counseling sessions and brief advice as 5–10 min brief advice on quitting smoking in combination with useful information on quiting (Brown et al., 2003). This brief advice is what is at times referred to as usual care because study staff give advice to smokers to quit in addition to provided quit smoking pamphlets (Hickman et al., 2015). MI is generally defined as client centered techniques that increase motivation especially among patients or clients with substance use disorders and lack the motivation to change their behavior (Brown et al., 2003). MI is said to target cognitive processes and help the client evaluate the perceived behavioral costs and benefits of changing behavior (Bandura, 1977, Brown et al., 2003, Velicer et al., 1985) MI is reported to be effective in increasing the readiness of changing behavior (Brown et al., 2003, Dunn et al., 2001).

Smokers with substance use disorders (SUD) have lower motivation to quit, a predictor of low success for smoking cessation (Flach and Diener, 2004, Martin et al., 2006, Richter et al., 2001, Rohsenow et al., 2015). In this group of smokers therefore, treatment methods such as motivational interviewing (MI) or brief advice (BA) that are geared to increase motivation are very much needed (Christie and Channon, 2014, Rohsenow et al., 2015). In one study, researchers found that there was no significant differences between MI and BA for a 7-day abstinence from smoking for follow ups within 1, 3, 6, and 12 months periods (Rohsenow et al., 2014). These same researchers reported that while the difference between MI and BA is minimal, BA might be a better choice for alcoholics in SUD treatment because of its strong authoritative nature compared to MI’s softer therapeutic and choice guided approach (Coleman, 2004, Miller and Rollnick, 2009, Rohsenow et al., 2014). In smokers with SUD, contingent vouchers (cv) was found to significantly increase smoking abstinence only during the period that incentives were available, and such increase dropped soon following termination of the incentives (Alessi and Petry, 2014, Alessi et al., 2008, Dunn et al., 2008, Dunn et al., 2010, Rohsenow et al., 2015).

### 5.4 Pharmacological strategies

Pharmacological interventions are divided into two categories, the nicotine replacement therapy (NRT), and the non-nicotine medications. There are 5 NRT products approved by the US Food and Drug Administration (FDA) and they include: nicotine patches, nicotine gum, nicotine nasal spray, nicotine lozenges, and nicotine vapor inhaler (Hurt, Ebbert, Hays, & McFadden, 2009). The FDA approved non-nicotine medicines are, bupropion, and varenicline (Hurt et al., 2009). In patients with schizophrenia, varenicline has been used and found to significantly decrease cotinine levels (p < 0.01), and objective or subjective measures of smoking (p < 0.01) in those patients who were smokers (Smith et al., 2016). All NRT products and medicines can be obtained in different doses for example the NRT patch comes in 21, 14, and 7 mg (Murphy et al., 2017), and nicotine gum in 6, 4, and 2 mg (Hansson, Rasmussen, & Kraiczi, 2017). While there was a dose effect of NRT patches among the general population of smokers, a higher dose of NRT (31.2 mg) did not produce significant differences compared to a lower dose (20.8 mg) among patients with schizophrenia or schizoaffective disorders (Chen et al., 2013, Fredrickson et al., 1995). As shown in the reviewed studies, some of the cessation strategies described in this paper and used in the general population are same as those in the psychiatric inpatient settings. The NRT dose effect example described here is however some of the evidence of a need for evidence-based interventions tailored to this population of psychiatric inpatients to help them quit smoking.

### 5.5 Gaps/challenges

This population of psychiatric inpatients who smoke have some of the greatest disparities associated with cigarette smoking yet we found only eight randomized control trial studies. This calls for additional RCT research in this area. Clinical trial research of smoking cessation is rare among individuals with psychiatric illness, and even the limited research has been mainly among outpatients and left out inpatients (Chen et al., 2013).

The limited research available shows that individuals with psychiatric illness experience the greatest exclusion in studies of smoking cessation (Kamholz et al., 2009, Le Strat et al., 2011). A critical issue deserving additional research and likely to improve cessation treatment among this population is an investigation of the appropriate timing to begin cessation treatment following a patient’s inpatient admission (Duffy et al., 2015). With the current trend of psychiatric hospitals going smoke free (Lawn and Campion, 2013, Soyster et al., 2016), there is likely an increase of psychiatric inpatients experiencing nicotine withdrawal symptoms (Soyster et al., 2016). Some of the symptoms of nicotine withdrawal are severe and might include increased feelings of anger, anxiety, restlessness, depressed mood, sleep disturbances, increased appetite, difficulty concentrating and craving (Hughes and Hatsukami, 1986, Soyster et al., 2016). These symptoms generally occur in the first 24 h of nicotine deprivation and severe in the first week of abstinence (Hughes, 2007, Soyster et al., 2016). There is need for an investigation of the effect of these symptoms in the general health of this population and their overall success in quitting smoking. Studies have indicated that the cessation treatments used among the mentally ill patients are the same as those among the general population yet individuals with mental illness are a unique population deserving of unique interventions (Peckham et al., 2017), or intensive treatment approaches (Marynak et al., 2018). In comparison other medically ill patients who achieve short-term abstinence while inpatients have increased success for quitting yet for those with behavioral or mental health diagnoses the cessation likelihood is low (Borrelli et al., 2010, Leeman et al., 2008, Ylioja et al., 2017).

## 6 Conclusion

Smoking cessation in inpatient settings is rare or delayed for reasons such as lack of knowledge of associations between smoking and psychiatric disorders. Although retention in smoking cessation studies can be difficult, psychiatric inpatient smokers can be engaged in cessation studies. Quitting smoking is difficult for individuals with psychiatric illness but they are just as interested in quitting as the general population and in some cases show quit attempts like those of the general population. While there was a dose effect of NRT patches among the general population of smokers, higher dose of NRT did not produce significant differences compared to a lower NRT dose among patients with schizophrenia or schizoaffective disorders. Tailored interventions with consideration of psychiatric inpatient specific needs are needed and may be helpful for psychiatric patients to quit smoking.

## Role of funding source

This work did not receive any specific grant from funding agencies in the public, commercial, or not-for-profit sectors.

## Contributors

Dr. Kagabo, the corresponding author wrote the first draft, performed literature review searches, and was the main contributor to the manuscript organization. Dr. Kagabo was supervised by Dr. Okuyemi. All the other authors contributed to the review, organization, and proof reading of the manuscript.

## Declaration of Competing Interest

The authors declared that there is no conflict of interest.
